# Oral mycobiome identification in atopic dermatitis, leukemia, and HIV patients – a systematic review

**DOI:** 10.1080/20002297.2020.1807179

**Published:** 2020-08-17

**Authors:** Camila Stofella Sodré, Paulo Matheus Guerra Rodrigues, Mayra Stambovsky Vieira, Alexandre Marques Paes da Silva, Lucio Souza Gonçalves, Marcia Gonçalves Ribeiro, Dennis de Carvalho Ferreira

**Affiliations:** a Faculty of Medicine, Department of Clinical Medicine, Universidade Federal do Rio de Janeiro - UFRJ, Rio de Janeiro, Brazil; b Laboratory of Oral and Systemic Infections, Faculty of Dentistry, Estácio de Sá University- UNESA, Rio de Janeiro, Brazil; c Faculty of Dentistry, Veiga de Almeida University- UVA, Rio de Janeiro, Brazil; d Medical Genetics Service, Martagão Gesteira Pediatric Institute (IPPMG- UFRJ), Universidade Federal do Rio de Janeiro-UFRJ, Rio de Janeiro, Brazil

**Keywords:** Mycobiome, fungi, HIV, leukemia, atopic dermatitis.

## Abstract

**Introduction:**

Oral mycobiome profiling is important to understand host–pathogen interactions that occur in various diseases. Invasive fungal infections are particularly relevant for patients who have received chemotherapy and for those who have HIV infection. In addition, changes in fungal microbiota are associated with the worsening of chronic conditions like atopic dermatitis (AD). This work aims, through a systematic review, to analyze the methods used in previous studies to identify oral fungi and their most frequent species in patients with the following conditions: HIV infection, leukemia, and atopic dermatitis.

**Methods:**

A literature search was performed on several different databases. Inclusion criteria were: written in English or Portuguese; published between September 2009 and September 2019; analyzed oral fungi of HIV-infected, leukemia, or AD patients.

**Results:**

21 studies were included and the most identified species was *Candida*. The predominant methods of identification were morphological (13/21) and sugar fermentation and assimilation tests (11/21). Polymerase chain reaction (PCR) was the most used molecular method (8/21) followed by sequencing techniques (3/21).

**Conclusions:**

Although morphological and biochemical tests are still used, they are associated with high-throughput sequencing techniques, due to their accuracy and time saving for profiling the predominant species in oral mycobiome.

## Introduction

Fungi are eukaryotes that are dispersed across various ecosystems and terrestrial environments, totalizing a seven-digit number of species [1]. However, estimates suggest that only 600 species are capable of causing disease in humans [2]. Nevertheless, invasive fungal infections are still underestimated both in the field of research and procedural guidelines by health agencies around the world [2]. Their epidemiology is still little understood due to the scarcity of studies, despite the fact that several fungal diseases such as malaria and tuberculosis are lethal [2]. Around 1.6 million people die each year from fungal infections [3], which are more challenging than infections by other microorganisms, in terms of the effectiveness of medicines available and time frame for administration [4]. In addition, several anthropogenic factors contribute to the appearance of specific and resistant forms [5], as evidenced by outbreaks of *Candida auris* in hospital settings [4,6].

The oral cavity is considered an important gateway for microorganisms (bacteria, fungi and viruses) that may be associated with localized oral problems such as periodontitis and candidiasis, or systemic diseases, including respiratory infections and cancer [7]. The oral cavity mycobiome consists of somewhere around 100 fungal species, where each individual presents an average of 9 to 23 species and 75 different genera [8]. Included among the most frequent genera are *Candida, Cladosporium, Aureobasidium, Aspergillus* and *Malassezia* spp., which are normally present in 25–75% of individuals [9]. Oral fungal infections are usually caused by *Candida* species, especially *C. albicans*, which is found to be the most prevalent and pathogenic due to its adherence ability, to form germ tubes and produce extracellular proteases [10–13]. These microorganisms are of great importance to oral health, as they can stimulate the immune system in a very different way to bacteria. Furthermore, they have the ability to form filamentous hyphae, which provide a support system for the formation of biofilms between fungal and bacteria species [14]. In the consortium of oral microbiota, in addition to fungal species, there are about 600 species of bacteria that are intrinsically associated with a person’s immune status. Dysbiosis is observed in several diseases such as infections by *Candida* spp. (oral candidiasis) and by *Aspergillus fumigatus* [15]. The incidence and severity of these infections, as well as the causative pathogens, is closely related to several factors, such as immune status, geographic location, and underlying diseases [16].

Accumulative evidence, since the first acquired immunodeficiency syndrome (AIDS) cases were reported in the 1980s, shows that patients infected with human immunodeficiency virus (HIV) are more susceptible to invasive fungal infections (IFI) [14,17]. HIV-infected patients present anti-fungal immune response failure, due to the effects of the virus in the lymphocytoid and myeloid cell lineages and the innate and adaptive response [17]. HIV impairs the immune system by depleting CD4+ memory T-cells, and this reduces the effector T-cell population [18]. HIV-infected patients are known to present lower Th2 cytokines levels than healthy subjects, and this is associated with *Pneumocystis* pneumonia [19], and the impairment of tumor necrosis factor-α (TNF-α) and interferon-γ in response to cryptococcal meningitis [17]. Despite their susceptibility to IFI caused by acquired fungi, several findings suggest that the HIV infection promotes a shift in fungal commensal microbiota that can lead to other infections. Oropharyngeal candidiasis (OPC) is the most common opportunistic disease found in the oral cavity and throat of HIV-infected patients, and it is strongly associated with CD4 + T-cells counts below 200 cells/mm^3^ [17,20,21]. Various studies have demonstrated that OPC symptoms can be ameliorated by anti-retroviral therapy (ART), and this is associated with immune restoring by viral load reduction and regeneration of CD4 + T-cells [20–22]. Interestingly, the effect of ART on OPC does not seem to be related to *Candida* colonization but rather to the development of an inflammatory response towards microbial antigens due to immune reconstitution [20,23]. This suggests that oral fungal microbiota could be modulated in such patients both by the causative pathogen (HIV) and its treatment. OPC in HIV-infected individuals is not only caused by *Candida albicans*, but also other species from this genus such as *Candida glabrata* and *Candida tropicalis*, which have been isolated in various studies [20].

IFI also represents a significative factor of morbidity and mortality for acute leukemia (AL) patients [24]. The increased risk of IFI in these patients relies on a combination of several factors related to the disease itself, the status of the patients’ health and the treatment modality [25]. In general, AL is characterized by an abnormal proliferation and differentiation of bone marrow progenitor cells that originate the myeloid and lymphoid cell lineages [25]. Acute myeloid leukemia (AML) leads to neutropenia and impaired granulocyte functions, and immature myeloid cells can inhibit T-cell specific antigen response [25]. On the other hand, acute lymphocytic leukemia (ALL) is characterized by the proliferation of poorly differentiated lymphoid cells [26]. Neutrophil-depletion is believed to be the main factor that predicts a higher risk of IFI in AML than in ALL, with exception of *Pneumocystis carinii* pneumonia that is more common in the latter [24,25,27]. Despite of these differences, AML and ALL treatments have high doses of cytotoxic drugs in common, which results in leukopenia [25]. In addition, radio- and chemotherapy injure the mucosal barrier and this creates a propitious entrance for opportunistic pathogens [25]. The most commonly isolated fungi from AL patients are *Candida, Fusarium, Aspergillus, Tricosporum,* and *Pneumocystis* [25]. There is evidence that highly cytotoxic drugs also promote dysbiosis in commensal microbiota, as seen in the gut of lymphoma patients that received myeloablative treatment pre-hematopoietic stem cell transplantation [28]. A recent longitudinal cohort study demonstrated that oral mycobiome is affected by remission-induction chemotherapy in AML patients, and that bacterial infection outcomes are related to modulations in *Candida* and *Fusarium* populations [29].

Fungal dysbiosis is particularly relevant in chronic conditions other than HIV-infection and is also related to immune imbalance. Atopic dermatitis (AD) is an inflammatory skin condition in which patients present overreaction to harmless antigens in both topic and airborne forms (mainly in adults) and also allergy to some types of food (especially in children) [30]. AD pathogenesis is believed to be mediated by deregulation of adaptive and innate responses associated with the disruption of the cutaneous barrier [30]. AD patients present abnormal levels of Th2 cytokines that is observed by increase of IL-4 and IgE levels, causing an immediate hypersensitivity reaction [31]. The progression of AD is related to fungi diversity. AD patients may have *Malassezia* on their skin and this can play a role in worsening AD patients’ conditions [32]. Even though most AD symptoms manifest themselves in the skin, AD exacerbation and attenuation are also related to *Candida* in other body sites [32]. *Candida albicans* was cultured from the nasopharynx of 59% of AD patients and its IgE-production stimulation was positively correlated to the severity of the disease [33]. Although the major studies in atopic dermatitis concern fungal characterization in the skin, the oral cavity presents a similar architecture but is rarely investigated [34,35]. In a sectional study, AD patients presented greater colonization by *Candida albicans* in the mouth when compared to healthy controls [36]. Interestingly, these patients presented lower serum levels of IgG against this species, which indicates alterations in the oral fungal microbiota [36]. Fungal microbiota in AD can be affected by therapy modalities. Outpatients that present moderate and severe forms of AD are in general treated with immunomodulators and corticosteroids, the latter being related to oral candidiasis and favoring cariogenic activity [34].

This body of evidence strongly suggests that fungal colonization, diversity, and interactions in oral microbiota are relevant for clinical outcomes of immune-mediated diseases, even though for those that have completely different etiologies. Moreover, these diseases often require hospitalization, which favors further colonization by opportunistic fungi [33]. Nosocomial fungal infections are difficult to diagnose and the occurrence of dysbiosis in these patients can aggravate their clinical condition, therefore, the techniques used to identify these microorganisms must become faster and more accurate [33,36].

Despite the advances made in recent years, oral mycobiome is still a poorly understood subject. As a public health issue, mycoses are classically diagnosed through morphological analysis in cultures [37,38], and often the definitive conclusion is only possible in post-mortem exams [24]. However, there are specific fungal stains that are effective for various species, such as *Aspergillus* spp., *Candida* spp., *Coccidioides immitis, Blastomyces dermatitidis,* and *Sporothrix schenckii* [38]. Non-culture-based tests include immunodiffusion (ID), enzyme immunoassays (EIA) and the complement fixation test [38]. Invasive aspergillosis can be detected by serum antigens such as galactomannan and 1,3-βD-glucan [38]. One of the most reliable serological tests involves the detection of *Cryptococcus neoformans* antigens in cryptococcal meningitis [38,39]. However, in medical mycology the most promising diagnostic field is that of molecular tools [39]. Methods involving polymerase chain reaction (PCR), mass spectrometry (MS) and sequencing are currently at the heart of the diagnostic revolution for invasive fungal infections [4,38].

The mycobiome field is underexploited and there is a bias due to the methods used for its characterization. Amplicon sequencing shows a lack of standardized methods by using different primers that can generate very distinguishable results [40]. Usually, studies focus on analyzing mycobiome in a specific subject or when studying a bacteria-fungi consortium. Therefore, identification techniques must be improved so that fungal taxonomic classification can become more reliable [41].

In order to understand the relevance of different mycobiome identification techniques, and their use in diseases that can react or perform differently in each individual, this systematic review selected three examples of diseases that are immune-mediated, present dysbiosis and are susceptible to nosocomial fungal infections: HIV-infection, leukemia, and atopic dermatitis. This study aims to identify the most prevalent fungal species in those diseases and the most affected sites. Additionally, this study aims to identify the more commonly used methods to identify the most prevalent fungi in the oral cavity of patients with these diseases.

## Materials and methods

The present study is a systematic review. It was performed in agreement with the rules of PRISMA (Preferred Reporting Items for Systematic Reviews and Meta-Analyses) [42] and was previously registered in PROSPERO under the protocol number CRD42020144972. In order to meet all the objectives proposed in this work, the following guiding questions were formulated for the preparation of this review: 1) What are the most common fungal species found in each disease? Is there a predominant species?/2) What were the sites of the oral cavity where the majority of the fungal species were isolated?/and 3) What are the most frequently used methods to identify fungi in the oral cavity in patients with one of these three diseases?

### Search strategy

**Figure 1.**
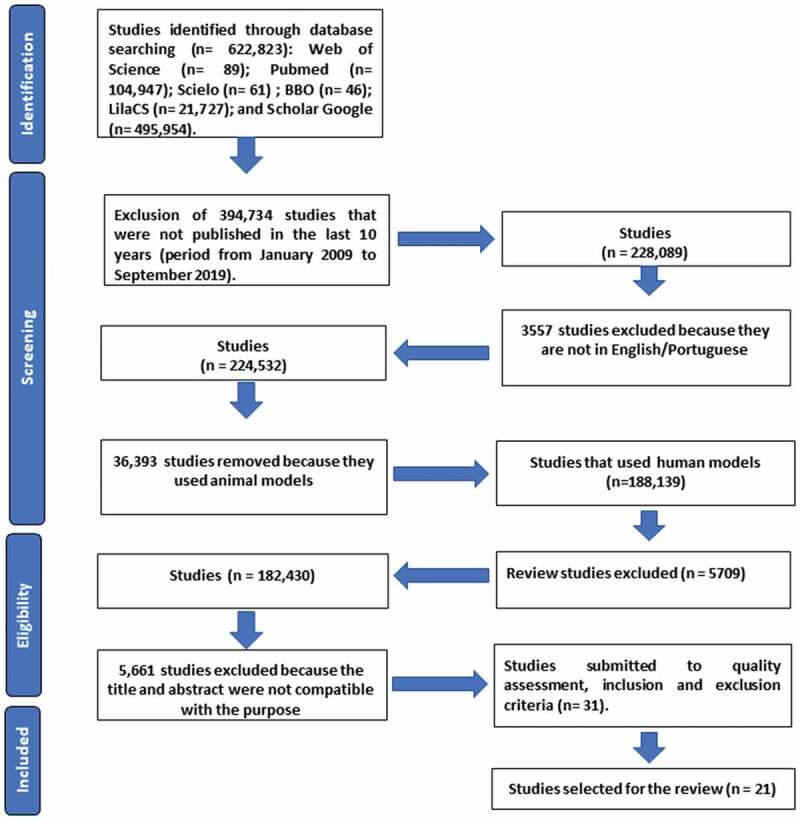
**Flow chart of selection process**. Database search was performed from January 2009 to September 2019.

A flow chart (see Figure 1) was used to select the articles in this bibliographic survey in three online databases in the health field: MEDLINE, Web of Science and Latin American and Caribbean Health Sciences (LilaCS) and four portals were also included in the search: PubMed which belongs to MEDLINE, Scientific Electronic Library Online (SciELO), Google Scholar, and Brazilian Library of Dentistry (BBO). The search was performed using the Boolean operators (AND, OR and NOT) and combined with the keywords previously checked by MEDLINE MeSH described below: ‘fungi’, ‘mouth’, ‘microbiota’, ‘atopic dermatitis’, ‘HIV’, ‘mass spectrometry’, ‘leukemia’, ‘mycobiome’, ‘18S’. These words were selected because of their relationship to the topic of interest. In addition, the term ‘fungi identification’ was used despite not being described in MeSH. The inclusion criteria included, original articles published in the last 10 years (period from January 2009 to September 2019); works published in full in the following languages: Portuguese and/or English and research with compatible titles and abstracts related to the topic of interest. The exclusion criteria were: articles whose theme was not related to the objective of the study; articles that used a language other than those accepted by the inclusion criteria and those that used an animal model, as well as theses, books, dissertations, patents, and literature and/or systematic reviews. After finalizing the inclusion and exclusion criteria, the quality assessment was performed for further descriptive analysis of the selected studies.

### Populations, Interventions, Control, Outcomes (PICOs)

We used the following PICOs criteria in our systematic review. Populations: leukemia, HIV-infected and atopic dermatitis patients who have been evaluated for the presence of fungal species in sites of their oral cavity. These patients may or may not have fungal lesion sites. Interventions: the present study will evaluate the methods used to identify fungal species in HIV-infected, atopic dermatitis and leukemia patients and thus verify the efficiency of each one. Control: healthy individuals with no fungal lesions in the oral cavity. Outcomes: 1) Identify and detail the main fungal profiling methods used for HIV, leukemia, and atopic dermatitis patients. 2) Identify the predominant fungal species found in HIV, leukemia, and atopic dermatitis patients. 3) Compare oral mycobiome between HIV, leukemia, and atopic dermatitis patients with control patients. 4) Identify the oral sites of patients with atopic dermatitis, leukemia, and HIV in which there is a predominance of isolated fungal species.

### Study selection and quality assessment

Two independent reviewers selected the studies and cross checked the keywords previously checked by MEDLINE MeSH. After, these two reviewers analyzed the titles and abstracts of each study independently. Duplicates were excluded and studies that were selected had their full-text analyzed by the two reviewers. If any disagreement occurred, a third reviewer was consulted. Data were summarized and the studies that met the inclusion and exclusion criteria had their methodology analyzed through quality assessment (Table 1) by the two reviewers. The quality assessment was based on the Preferred Reporting Items for Systematic Reviews and Meta-Analyses (PRISMA) criteria and established by the two reviewers (C.S.S and P.M.G.R.). Each item of the quality assessment presented a score of 1 point. Studies were classified as level A (high methodological quality) if they received scores between 7 and 9 indicates. If they scored between 5 and 6 points were classified as level B (intermediate methodological quality). Studies that were classified as level A and B were included in this study, and if they scored below or equal to 4 points (level C- low methodological quality) were excluded.

**Table 1.** Quality assessment of the selected studies for the systematic review.

ITEM	QUALITY ASSESSMENT	YES	NO
1	Representative sample of the population	1	0
2	Definition of study type	1	0
3	Definition of inclusion and exclusion criteria for the target population	1	0
4	Presence of control group	1	0
5	Colorimetric and morphological identification of fungal species	1	0
6	Identification of species by biochemical tests for sugar fermentation and assimilation and/or mass spectrometry	1	0
7	Identification of fungal species by PCR and molecular analysis	1	0
8	Statistical analysis	1	0
9	Discussion of study limitations	1	0

## Results

In the initial search (Figure 1), a total of 622,823 studies were found in 2 portals and 4 databases: Web of Science (n = 89); PubMed (n = 104,947); SciELO (n = 61); BBO (n = 46); LilaCS (n = 21,727); and Google Scholar (n = 495,954). After the inclusion and exclusion criteria were applied, there were a total of 49 studies, and after their duplicates had been removed, there were 31 articles left. Then, the quality assessment was carried out, which studies that were classified with high and intermediate methodological quality were selected. After the quality assessment, 21 studies were selected for this systematic review (Table 1).

### Characteristics of selected articles

Among the 21 studies, 11 defined their type of study as: cohort (6/11); prospective (3/11); and transversal (2/11). All studies were in English and showed a great variability in the number of samples, ranging from 24 to 990 patients. Most of the studies addressed HIV-infection, with 18 studies in total, while atopic dermatitis (1/21) and leukemia (2/21) were minorities. Also, most of the studies used adult patients (16/21), however, five articles evaluated pediatric patients: three studies involving HIV-infected patients and the two studies that evaluated leukemia patients. Another aspect observed was the method to collect the clinical specimens: the oral swab (9/21) and the oral rinse (6/21) were the most commonly used methods to collect from the oral cavity. The oral rinse can evaluate not only the *Candida* load but also detect other microorganisms, while the oral swab is more suitable for specific sites of microbiological examination [43].

The identification of the species showed that most studies identified *Candida* species, especially *C. albicans* (12/21), *C. tropicalis* (13/21), *C. parapsilosis* (12/21) and *C. dubliniensis* (11/21). Articles that identified fungi in HIV patients also found fungi genera other than *Candida*, such as: *Malassezia, Debaryomyces,* and *Aspergillus* [44–47]. The description of the selected studies is given in Table 2. The species identified in each study and their main objectives are described in Table 3.

**Table 2.** Description of studies selected by the quality assessment.

Authors, country and year	Databases	Keywords	Disease studied Type of study	Sample size	Sample type	Quality Assesment
Das Chagas et al. Brazil 2009 [50]	Pubmed	Fungi; mouth; HIV	Human immunodeficiency syndrome (HIV)	30 children with HIV	Oral swab and decayed dentin	B
			Does not mention the type of study			
Junqueira et al. Brazil 2012 [51]	LilaCS	Fungi; HIV	Human immunodeficiency syndrome (HIV)	60 soropositive patients with oropharyngeal candidacy	Oral swab from the Candida injury site, and Saliva	B
			Does not mention the type of study			
Mane et al. India 2012 [70]	LilaCS	Fungi; HIV	Human immunodeficiency syndrome (HIV)	335 patients: 210 HIV-positive 125 HIV-negative	Oral swab	A
			Prospective study			
De Mendonça et al. Brazil 2012 [62]	Web of Science	Fungi; microbiota; leukemia	Leukemia	71 pediatric patients	Saliva	B
			Cohort study			
Moris et al. Brazil 2012 [52]	LilaCS	Fungi; HIV	Human immunodeficiency syndrome (HIV)	214 HIV-positive patients	Oral swab	B
			Does not mention the type of study			
Dos Santos Abrantes et al. South Africa and Cameroon 2013 [54]	LilaCS	Fungi; HIV	Human immunodeficiency syndrome (HIV)	212 HIV-positive patients 262 HIV-negative patients	Oral swab	B
			Does not mention the type of study			
Merenstein et al. USA 2013 [55]	LilaCS	Fungi; HIV	Human immunodeficiency syndrome (HIV)	84 women: 59 HIV-positive women 25 HIV-negative women	Oral swab	A
			Non-interventional prospective cohort study			
Li YY et al. China 2013 [47]	LilaCS	Fungi; mycobiome; HIV	Human immunodeficiency syndrome (HIV)	604 HIV-positive patients 851 HIV-negative individuals	Oral tongue and mucosa swab	A
Sharifzadeh et al. Iran 2013 [56]	Pubmed	Fungi; HIV	Human immunodeficiency syndrome (HIV)	100 HIV-positive patients	Oral tongue swab, oral mucosa and lesions	B
	LilaCS	Fungi; microbiota; HIV	Does not mention the type of study			
Jiang et al. China 2014 [57]	Pubmed	Fungi; mouth; HIV	Human immunodeficiency syndrome (HIV)	45 HIV- positive adult patients 31 systemically healthy individuals	Oral rinses	A
	LilaCS	Fungi; HIV	Prospective study			
Mukherjee et al. USA 2014 [44]	Pubmed	Fungi; HIV	Human immunodeficiency syndrome (HIV)	12 HIV-positive patients 12 systemically healthy individuals	Oral rinses	B
	Web of Science	Fungi; mouth; microbiota; HIV	Does not mention the type of study			
Thanyasrisung et al. Thailand 2014 [58]	LilaCS	Fungi; HIV	Human immunodeficiency syndrome (HIV)	60 HIV-patients 49 systemically healthy individuals	Oral rinses	A
			Does not mention the type of study			
Javad et al. Iran 2015 [36]	Pubmed	Atopic dermatitis; Fungi	Atopic dermatitis (AD)	100 patients with AD 50 systemically healthy individuals	Oral swab and scalpel	B
			Does not mention the type of study			
Blignaut et al. South Africa 2015 [72]	Pubmed	Fungi; HIV	Human immunodeficiency syndrome (HIV)	362 HIV-positive children	Tongue and dentine swab	A
	LilaCS	Fungi; mouth; HIV	Cohort study			
Menezes et al. Brazil 2015 [59]	Pubmed	Fungi; HIV	Human immunodeficiency syndrome (HIV)	147 HIV-positive patients	Unstimulated saliva	B
			Cross-sectional study			
De Mendonça et al. Brazil 2015 [61]	Pubmed	Fungi; microbiota; leukemia	Acute lymphoblastic leukemia (ALL)	71 pediatric patients with ALL aged between 3 and 276 months	Oral mucosa material and blood sample	B
	Web of Science		Cohort study			
Mushi et al. Tanzania 2016 [73]	Pubmed	Fungi; mouth; HIV	Human immunodeficiency syndrome (HIV))	351 HIV-positive patients 639 HIV-negative patients	Oral rinses	B
			Transverse cohort study			
Lourenço et al. Brazil 2017 [60]	Pubmed	Fungi; mouth; HIV	Human immunodeficiency syndrome (HIV)	25 HIV-negative patients 48 HIV-positive patients	Oral rinses	A
			Does not mention the type of study			
Fukui et al. Japan 2018 [45]	Pubmed	Fungi; mycobiome; HIV	Human immunodeficiency syndrome (HIV))	46 HIV-positive patients 20 HIV-negative patients	Palatine tonsil swab	A
			Cross-sectional study			
Mukherjee et al. USA 2018 [53]	Pubmed	Fungi; HIV Fungi; mouth; microbiota; HIV Fungi; mycobiome; HIV	Human immunodeficiency syndrome (HIV)	24 HIV-negative individuals 72 HIV-positive patients	Oral rinses	A
	Web of Science		Cohort study			
Vijendran et al. India 2018 [46]	Pubmed	Fungi; HIV	Human immunodeficiency syndrome (HIV)	100 HIV-positive patients 100 HIV-negative patients	Tongue-dorsum swab	A
	Google Scholar		Prospective study

**Table 3.** Isolated species and study objectives.

Authors, country and year	Goals	Main outcome	Identification method	Fungi species isolated
Das Chagas et al. Brazil 2009 [50]	Evaluate oral mucosal Candida species of children with HIV before and after dental treatment.	After dental treatment, there was a reduction of Candida in the oral mucosa.	Colorimetric and morphological identification and sugar fermentation and assimilation tests	*C. albicans* *C. tropicalis* *C. parapsilosis*
Junqueira et al. Brazil 2012 [51]	To evaluate the presence of pathogenic yeasts of saliva and oropharyngeal candidiasis in HIV-positive patients	Brazilian HIV patients are colonized and infected with yeasts composed of different species such as Candida, non-albicans species and other species	Sugar fermentation and assimilation tests (API20 C); and morphological tests: germ-tube test, hyphae production, cornmeal agar chlamydospore	*C. albicans* *C. dubliniensis*
Mane et al. India 2012 [70]	To determine the biofilm production of Candida isolates from HIV-positive and HIV-negative patients	There was an improvement in the ability to form oral Candida isolates from HIV-positive individuals compared to non-infected individuals	Sugar fermentation and assimilation tests (API 20AUX)	*C. parapsilosis* *C. glabrata* *C. tropicalis* *C. krusei* *C. lusitaniae* *C. kefyr*
De Mendonça et al. Brazil 2012 [62]	To prospectively investigate associations between HSV-1; *Candida spp*; and oral bacteria with the oral mucositis severity	The presence of HSV-1 and Candida was associated to oral mucositis severity in all pediatric patients	Colorimetric and morphological identification	*C*. spp.
Moris et al. Brazil 2012 [52]	To distinguish species from a *C parapsilosis* complex isolated from HIV-positive individuals	*C metapsilosis* is a commensal fungus but its importance as a pathogen must be confirmed	Colorimetric and morphological identification and molecular techniques (PCR/RFLP)	*C. parapsilosis* *C. albicans* *C. glabrata* *C. tropicalis* *C. dubliniensis* *C. krusei*
Dos Santos Abrantes et al. South Africa and Cameroon 2013 [54]	To determine the prevalence of Candida species	Drug resistance is common in HIV patients from South Africa and Cameroon	Colorimetric and morphological identification; germ-tube test and gram coloring	*C. albicans* *C. glabrata* *C. tropicalis* *C. dubliniensis*
Merenstein et al. USA 2013 [55]	Compare the colonization proportions of oral and vaginal Candida in HIV-positive and negative patients.	Oral and vaginal *Candida* colonization was more frequent in HIV-positive participants than in HIV-negative controls.	Sugar fermentation and assimilation tests (API20 C); morphological tests: germ tube, culture media for chlamydospores production	*C. parapsilosis* *C. albicans* *C. glabrata* *C. tropicalis* *C. lusitaniae*
Li YY et al. China 2013 [47]	To investigate oral transport rate of asymptomatic *Candida*, its species distribution and fungal susceptibility.	Oral fungi colonization is associated with CD4+ cells count and retroviral therapy.	Colorimetric and morphological identification; sugar fermentation and assimilation tests (API20 C); morphological tests: germ tube, culture media for chlamydospores and xylose assimilation.	*C. glabrata*, *C. parapsilosis*, *C. krusei*, *C. tropicalis*, *C. rugosa*, *C. norvegensis*, *Pichia ohmeri Saccharomyces cerevisiae*
Sharifzadeh et al. Iran 2013 [56]	To evaluate prevalence of pathogenic organisms with oropharyngeal candidiasis-related *Candida* species in HIV-positive patients.	Oral *Candida* is not associated with CD4+ cells count except when the count is above 400 cells/µl	Molecular method (PCR) and morphological tests: germ tube, hyphae production and culture media for chlamydospores production	*C. parapsilosis* *C. albicans* *C. glabrata* *C. tropicalis* *C. dubliniensis* *C. krusei* *C. lusitaniae* *C. guilliermondii* *C. norvegensis* *Kluyveromyces* *Cr. albidus*
Jiang et al. China 2014 [57]	To investigate oral *Candida* presence, its distribution and anti-fungal susceptibility during the first year of antiretroviral therapy in HIV-positive adults.	Rise of CD4+ cells count is associated with less *Candida* presence.	Sugar fermentation and assimilation test (API20 C); morphological tests: germ tube, hyphae production and culture media for chlamydospores production	*C. parapsilosis* *C. albicans* *C. glabrata* *C. tropicalis* *C. dubliniensis* *C. krusei* *C. kefyr* *C. guilliermondii* *C. norvegensis* *C. rugosa* *C. sphaerica* *Rhodotorula glutinis*
Mukherjee et al. USA 2014 [44]	To identify main mycobiome and bacteriome in HIV-positive and HIV-negative individuals.	More *Candida* colonization was associated with less *Pichia* abundance.	Molecular methods (PCR) Pyrosequencing for mycobiome analysis (MTPS)	Mycobiome identification. Some identified genera were: *Candida, Aspergillus, Fusarium* and *Pichia*
Thanyasrisung et al. Thailand 2014 [58]	To determinate *Candida* species prevalence in HIV-positive adults.	*Candida* colonization is influenced by immune status, tuberculosis presence and contraceptive use	Colorimetric and morphological identification; sugar fermentation and assimilation test (API20 C); morphological test: culture media for chlamydospores production; molecular method (PCR)	*C. parapsilosis* *C. glabrata* *C. tropicalis* *C. dubliniensis* *C. krusei* *C. lusitaniae*
Javad et al. Iran 2015 [36]	To verify *Candida* colonization and to verify humoral-specific response against *C. albicans* in AD patients.	IgG-levels in sera from AD patients were significantly lower than in controls.	Colorimetric and morphological identification; molecular method (PCR)	*C. parapsilosis* *C. albicans* *C. glabrata* *C. dubliniensis*
Blignaut et al. South Africa 2015 [72]	To demonstrate *C. albicans* presence in soft and hard tissues and to investigate by molecular techniques the genetic subtype from two different oral sites.	Decayed teeth can be considered potential *C. albicans* reservoirs, and they can cause infection in immunocompromised patients.	Colorimetric and morphological identification; morphological test: germ tube; sugar fermentation and assimilation test (ID32 C); molecular method (DGGE)	*C. albicans*
Menezes et al. Brazil 2015 [59]	To evaluate and quantify *Candida* species	Low CD4+ cells count is associated with high yeast density in HIV-positive patients.	Colorimetric and morphological identification; sugar fermentation and assimilation test (Auxacolor2 system); molecular method (PCR)	*C. parapsilosis* *C. albicans* *C. glabrata* *C. tropicalis* *C. dubliniensis* *C. krusei* *C. lusitaniae* *C. kefyr* *C. guilliermondii* *C. peliculosa* *C. famata*
De Mendonça et al. Brazil 2015 [61]	To investigate associations between oral microbiota, white cells, neutrophil and platelet counts and hemoglobin levels.	Presence of *Candida* species is associated with mucositis worsening in children and adolescents.	Morphological tests.	*Candida* spp.
Mushi et al. Tanzania 2016 [73]	To compare *Candida* non-*albicans* colonization in HIV-positive and HIV-negative individuals from Mwanza, Tanzania.	*Candida* non-*albicans* frequency is higher in HIV-positive individuals with a low CD4+ cell count that can facilitate invasive infections.	Colorimetric and morphological identification; mass spectrometry (MALDI-TOF MS)	*C. glabrata* *C. tropicalis* *C. dubliniensis* *C. krusei*
Lourenço et al. Brazil 2017 [60]	To verify periodontal effects in prevalence of *Candida* species.	Periodontal disease is associated with an increase in *Candida* species in HIV-positive patients.	Colorimetric and morphological identification; morphological tests: micro cultivated fungi test, hypertonic *Sabouraud* broth, germ tube test, tetrazolium reduction, hyphae production, culture media for chlamydospores production; sugar fermentation and assimilation tests: API (ID 32 C).	*C. parapsilosis* *C. albicans* *C. glabrata* *C. tropicalis* *C. dubliniensis* *C. krusei*
Fukui et al. Japan 2018 [45]	To characterize tonsil microbiome in HIV-positive patients.	There is dysbiosis in tonsil microbiome of HIV-positive patients in an immune status-independent manner.	DNA sequencing and bioinformatics.	*C*. spp. *Malassezia* *Saccharomyces* *Debaryomyces* *Aspergillus* *Penicillum*
Mukherjee et al. USA 2018 [53]	To verify a relationship between microbial dysbiosis, smoking and HIV markers.	Clinical and immune variables of HIV patients are related to oral microbiota dysbiosis	DNA sequencing and bioinformatics.	*C. parapsilosis* *C. albicans* *C. tropicalis* *C. dubliniensis* *C. lusitaniae* *C. rugosa* *C. orthopsilosis* *C. oxycetoniae* *C. versatilis* *C. ethanolica* *C*. spp.
Vijendran et al. India 2018 [46]	To verify clinical spectrum of surface mycoses, its microbial etiology and the antifungal-resistance pattern.	Isolated species from HIV-positive patients demonstrated increased resistance to antifungals.	Colorimetric and morphological identification; morphological tests: germ tube test, spore formation test; sugar fermentation and assimilation tests.	*Dermatophytes* *Pityrosporum* *Non-dermatophyte Candida* spp.

### Fungal colonization in HIV-infected patients

The oral mycobiome of HIV-infected individuals has been demonstrated to be different when compared with non-HIV-infected patients [48], and is most likely to be correlated with the immunodeficiency observed in HIV-infected individuals [49]. Distinct microbial communities in the digestive tract of HIV-infected individuals under antiretroviral therapy (ART) can be found; furthermore, patients with a history of AIDS, displaying T CD4+ lymphocytes count < 200 cells/mm^3^, have significantly lower subgingival plaque fungal diversity [49]. The interaction in both gut and oral bacterial diversity can be correlated with the use of specific ART regimens. For instance, the use of protease inhibitors (PI) with nucleoside reverse transcription inhibitors (NRTI) can be associated with decreased oral diversity compared to an NRTI + non-nucleoside reverse transcription inhibitors (NNRTI) regimen. In the gut, on the other hand, integrase strand transfer inhibitors (INSTI) combined with NRTIs can demonstrate significantly higher diversity compared to a combination of NRTI and NNRTI [49]. These imply long-term effects of HIV-infection on the mycobiome as well as the bacterial microbiome, which is either not completely mitigated by ART or represents effects of the treatment itself with disruptions of the gut barrier integrity [49].

The results of this review point to the importance of the genus *Candida* in the oral colonization of patients with HIV. *C. albicans* was present in 11 HIV studies selected in our review, as well as *C. tropicalis* which was found in 13 studies [44,47,50–60]. Although the presence of *Candida* spp. in the most of the articles that focused on HIV-infected patients, Mukherjee et al. [44], Mukherjee et al. [53] and Fukui et al. [45] through sequencing identified genera other than *Candida*. Mukherjee et al. [44] found differences between the HIV-infected mycobiome and uninfected individuals. The authors found a prevalence of *Candida, Epicoccum* and *Alternaria* in HIV-infected patients (92, 33, and 25%, respectively), while in uninfected individuals the most frequent genera were *Candida, Pichia* and *Fusarium* (58%, 33% and 33% respectively). The core of the oral mycobiome in both HIV-infected and uninfected individuals consisted of 5 genera of which only 2 (*Candida* and Penicillium) were common to both groups [44]. Mukherjee et al. [44] evaluated the correlation between different fungi that were presented in the mycobiome of HIV-infected patients and found 6 fungal–fungal interactions (*Candida-Epicoccuum, Candida-Trichosporon, Epicoccum-Trichosporon, Penicillium-Corynespora, Penicillium-Fusarium,* and *Alternaria-Serpula*) that were statistically significant (*p*≤ 0.03) [44]. Mukherjee et al. [53] analyzed the mycobiome of HIV-infected smoker and non-smoker patients using sequencing and bioinformatic analysis [53]. The Shannon diversity index pointed to a significantly low diversity in the mycobiome of HIV-infected non-smokers compared to HIV-infected smokers and uninfected smokers but not at a genus level (*p*≤ 0.02) [53]. The core mycobiome included 11 fungal genera in common in the three groups and 4 which were only detected in the HIV-infected smoker group (*Cladosporium, Nakaseomyces, Scleroderma*, and *Rhodotorula*) [53]. At species level, 2 were unique to the HIV-infected smoker group (*C. glabrata* and *Scleroderma* spp.). Levels of *Candida* were higher in the HIV-infected and uninfected smoker groups (34.7% and 38.45, respectively) compared to HIV-infected non-smoker group (19.2%). In summary, the fungal diversity of the mycobiome was significantly greater in HIV-infected smokers compared to HIV-infected non-smokers [53].

Fukui et al. [45] used amplicon sequencing to compare HIV-infected and non-infected individuals. There was no significant difference regarding the mycobiome of the palatine tonsils between HIV-infected and non-infected individuals. At the phylum level, Ascomycota and Basidiomycota were abundant but no significant difference was observed and at species level *Candida* and Malassezia colonized both groups [45].

### Fungal colonization in patients with leukemia

In the present study, only 2 articles that related to fungal identification in patients with leukemia were selected. De Mendonça et al. [61] and De Mendonça et al. [62] conducted their studies in pediatric patients with acute lymphoid leukemia (ALL) who were undergoing chemotherapy during collection. Both studies were published by the same study group. Their methodology included material collection on the 14th day after the start of chemotherapy, a severity assessment of mucositis in the patients and microbiological analyses.

In the second study, De Mendonça et al. [61] collected samples using mucosal swabs and found a significant association in their patients between the severity of mucositis with three variables: presence of herpes simplex virus type I (p = 0.0347), presence of *Candida* spp. (p = 0.0078) and low platelet count (p = 0.0064). While, in the first study De Mendonça et al. [61] collected samples via an oral rinse and also observed the same significant association between the severity of mucositis with the presence of the herpes simplex virus type I (p = 0.0347) and the presence of *Candida* spp. (p = 0.0078), but did not include hematological factors in their work. De Mendonça et al. [62] found that on the 14th day 25.4% of their patients were colonized by *Candida*. This value decreased to 14.9% of the patients on the 56th day. Although the authors did not refer to the possible causes of the observed reduction, they made it clear in their methodology that all patients received routine dental treatment during the study and that no antifungal agents were administered. In the second work of the group, there was a small reduction in the presence of *Candida* on the 56th day. The number of patients with severe mucositis appeared to be greater on the 14th day than on the 56th day.

### Fungal colonization in patients with atopic dermatitis

The colonization of the cutaneous microbiota of patients with AD is different from healthy individuals, with *Staphylococcus aureus* being the most commonly found microorganism [63–66]. This is propitiated due to defects in the function of the skin barrier and deficiency of ceramides that facilitate the penetration of microorganisms and allergens [63–66]. In atopic dermatitis, most studies are focused on the cutaneous microbiota, and few have verified the microbial oral status of these patients. Generally, the cutaneous mycobiome of patients with AD have *Malassezia* spp [32,67] and *Candida* spp [36].

After our selection and quality assessment, only one article was included in this survey. The results verified the colonization of *Candida* and the humoral immune response specifically against the species *C. albicans* in the skin and oral cavity [36]. This study found a higher frequency of colonization by *C. albicans* in patients than in controls (23% vs. 5%, p < 0.05), although there was no statistically significant difference in relation to colonization by the genus *Candida*. In general, the most isolated species were *C. albicans* and *C. glabrata*, both in the control group and in patients with AD. In another study reporting on the levels of immunoglobulins, Javad et al. [36] found that 14% of AD patients colonized by *Candida* had IgM levels below 10 U/ml, while specific IgG levels were significantly lower for *C. albicans* in patients than in controls [36]. This result may be linked to the fact that IgG plays a key role in the humoral immune response and may also be related to increased colonization of *C. albicans* in patients with AD [36,68].

### Fungal identification methods

**Figure 2.**
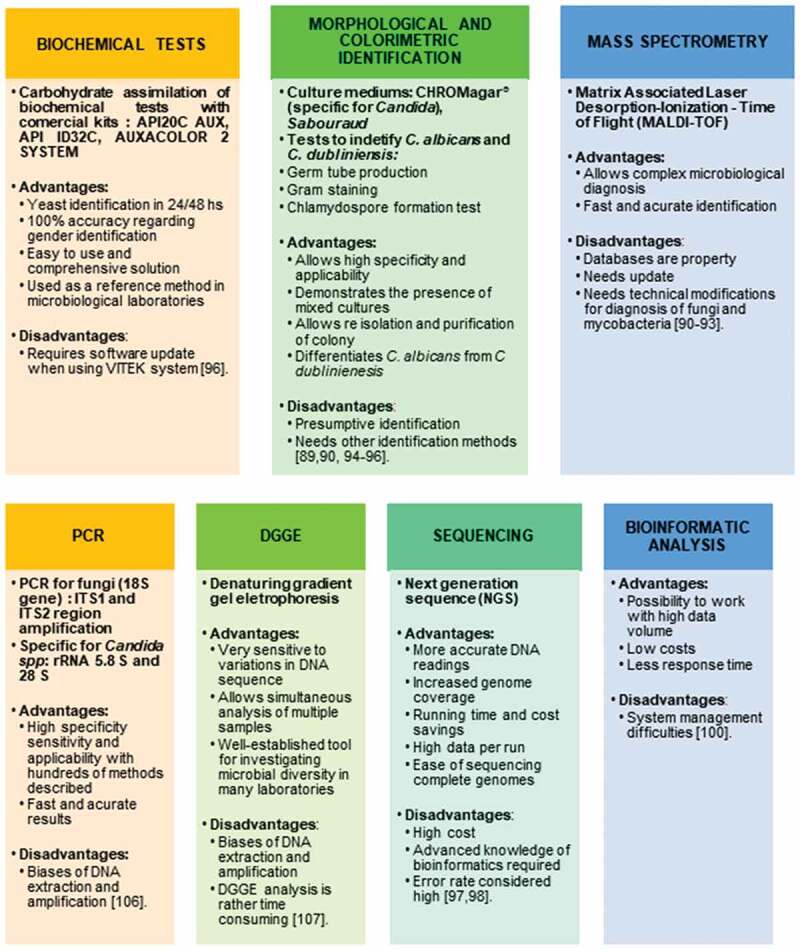
**Most frequent methods used to identify fungi**. Mycobiome identification can be divided into 7 general approaches, with each one presenting advantages and disadvantages.

This study describes different methods to identify fungi (Figure 2). Some are only presumptive, such as the colorimetric and morphological identification methods, and these were the most used technique in the selected articles (13/21), followed by sugar fermentation and assimilation tests (11/21), which are used specially to distinguish the different *Candida* species [69]. Among the molecular methods, the polymerase chain reaction (PCR) was the most used (8/21) in the selected articles. This result demonstrates an evolution compared to the previous identification methods linked to microscopic aspects such as: the germ tube method (9/21) and specific means for the production of chlamydospore (8/21), which are tests used as a standard to identify the species of *Candida albicans* [70]. Some studies did not use molecular techniques, but used biochemical tests to confirm the presumptive results of colorimetric and microscopic identification methods. Among these techniques, the most widely used are the sugar fermentation and assimilation tests, which currently have several commercial kits that have facilitated and disseminated their use in laboratories such as API20 C AUX (Biomérieux®), which is one of the most used commercial systems for the identification of yeasts [71]. The articles that used commercial kits to identify the most common *Candida* species were: Das Chagas et al. [50]; Junqueira et al. [51]; Mane et al. [70]; Merestein et al. [55]; Li et al. [47]; Jiang et al. [57]; Thanyasrisung et al. [58]; Blignaut and van Heerden [72]; Menezes et al. [59]; Lourenço et al. [60]; and Vijendran et al. [46]. Mushi et al. [73] was the only work that used MS focused on microbiology to identify fungi. They chose to perform MALDI-TOF spectrometry (‘Matrix Associated Laser Desorption-Ionization – Time of Flight’), which compares a mass spectrum of a microorganism that has been isolated with a spectrum of strains already known that are being used as references in the program library [62,63]. Blignaut and van Heerden [72] also evaluated the genetic subtype of *C. albicans* from two different oral sites (tongue swab and dentin scrape) by using the denaturing gradient gel electrophoresis technique, better known by the acronym DGGE after having identified the species by the commercial kit ID32 C (Biomérieux®).

The sequencing method was performed on three articles that evaluated HIV-infected patients [44,45,53] and later Fukui et al. [45] and Mukherjee et al. [53] did the analysis by bioinformatics. Mukherjee et al. [44] carried out pyrosequencing to analyze the microbiome and the characterization of nucleic acids, while Mukherjee et al. [53] used the Ion Torrent sequencing platform, which uses pH to identify the bases [74]. Fukui et al. [45] used the Illumina platform which, like the Sanger method, performs sequencing by synthesis of the DNA polymerase enzyme and terminator nucleotides labeled with different fluorophores [75].

**Figure 3.**
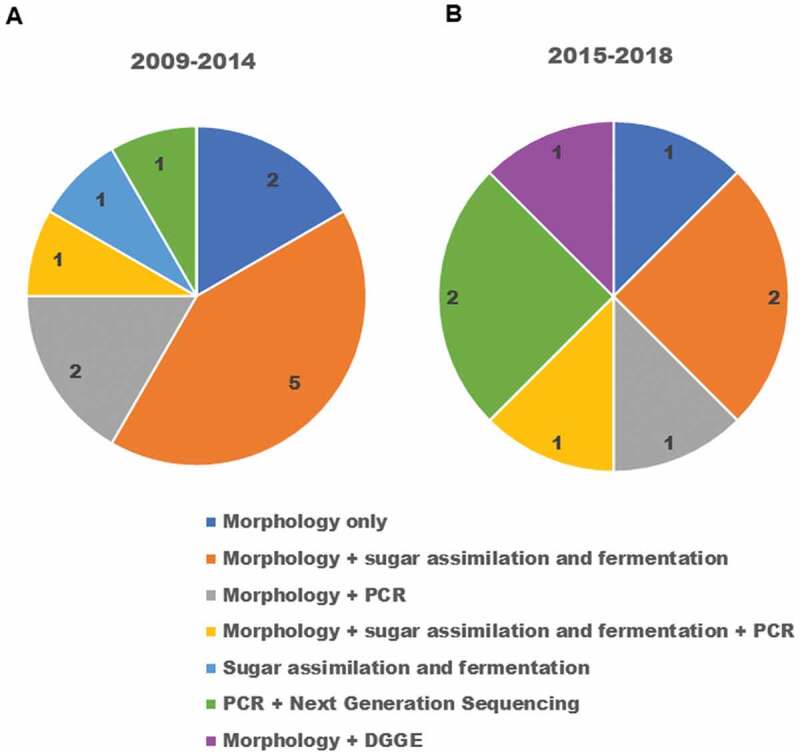
**Comparison of fungal profiling approaches by the selected studies**. (a) Identification methods used by studies published between years 2009–2014. (b) Identification methods used by studies published between years 2015–2018.

Over the years, the identification protocols have changed. Morphological methods are frequently being executed first and then the molecular approaches are applied next. Furthermore, this trend is not an exclusive feature of oral fungal identification but is a trend in the entire field of microbiology and its subset mycology [8,76–79]. Figure 3(a) shows that the morphological methods were the most used in the period from 2009 to 2014 and these were followed by the sugar fermentation and assimilation tests in the same period. However, in the period from 2015 to 2018 (Figure 3(b)) we can see that there is a balance between the morphological methods together with the sugar fermentation and assimilation tests and PCR and next-generation sequencing, which indicates the evolution of resources and diffusion of molecular techniques among research laboratories.

## Discussion

The role of fungi as opportunistic pathogens has gained prominence in recent decades, mainly due to the emergence of the AIDS pandemic in the 1980s [2]. Fungal infections are probably one of the main challenges for medicine in the 21^st^ century, as they have been neglected in recent decades compared to other pathogens, such as bacteria and protozoa [4]. Currently, there is a clear association between immune-mediated diseases and susceptibility to invasive fungal infections [80,81], which can lead to morbidity and clinical worsening of a patient’s health [82–84].The use of low cost and optimized methods to identify the genus and species of microorganisms in hospital environments are required so that they can be detected quickly and preventive measures can be taken against infection [82–84]. Conventional laboratory techniques using a microscope and culture medium require days until the microorganism is in the optimum growth phase and can be identified [85,86]. In addition, these techniques generate a high cost and the need for a team trained in a microbiology laboratory [85]. An alternative technique that can be used routinely for fungi diagnosis is MS with matrix-assisted laser ionization and desorption source and time-of-flight analyzer (MALDI-TOF), which performs the diagnosis in a short time and precisely [87–89]. Currently, this technique also performs the identification of filamentous fungi after undergoing adaptations in the extraction process to obtain protein profiles [89]. The use of morphological techniques for the identification of fungi is important to understand the evolution of morphological characteristics of fungi and to classify them according to their family and genus level [90,91]. However, this method is not very effective in identifying the species level, and is a challenge when performed by non-specialists [90,92]. Culture media are an example of morphological techniques that can be used to identify and distinguish species [93]. This study demonstrated that despite presenting limitations, morphological techniques such as colorimetric identification CHROMagar® as well as the germ tube test are still widely used prior to the molecular techniques [36,52,56,72]. In this case, molecular techniques were used after these morphological analyses in order to confirm the species identified at the molecular level because the mycological identification of species becomes necessary when the investigated patient is already immunocompromised [68].

In the present review, only the works of Mukherjee et al. [44], Mukherjee et al. [53], Fukui et al. [45], Li et al. [47] and Vijendran et al. [46] identified genera other than *Candida* in HIV-infected patients. This can be explained due to the techniques used by their studies: Mukherjee et al. [44] used the Multitag 454 Pyrosequencing; Mukherjee et al. [53] used the Ion-Torrent sequencing platform; and Fukui et al. [45] used the Illumina Miseq. Next-generation sequencing provides the identification and typing applications of all pathogens in a single protocol, which contrasts with Sanger sequencing [93]. This advantage is of great use in medical microbiology and also for preventive measures against infection [93]. However, next-generation sequencing requires basic knowledge in bioinformatics, which makes its use more difficult [94]. Thus, when comparing the advantages and differences between sequencing techniques, we can see that the Ion Torrent platform, used by Mukherjee et al. [53] is the simplest and also has a low cost, and consequently, it is widely available, especially in Europe and the USA [74]. The Multitag 454 Pyrosequencing that was used in the work of Mukherjee et al. [53] is more efficient than the Sanger method presenting greater sequence coverage capacity, precision, flexibility, parallel processing, easy automation potential, and instantaneous sequencing response [95]. However, its use requires the use of numerous reagents, in addition to presenting a high cost and an error rate that is considered high (0.0098) [96].

In relation to the sequencing platforms, there were some differences between the results, which may be linked to the platform used, as well as the distinct sample types. Fukui et al. [45] used palatine tonsil swabs while the other works used oral rinses. The Illumina sequencing platform was the most used platform in the selected studies that investigated fungi in HIV-patients, however, it has a higher cost and a long analysis time [80]. Fukui et al [45] showed, with the use of the Illumina platform that the fungal diversity and its composition in the HIV-infected and non-infected groups were the same [45]. In this work, the authors verified that the alpha and beta diversity of the mycobiome of the palatine tonsil between HIV-infected and non-HIV-infected patients did not have a significant difference. Unlike Fukui et al. [45], the work of Mukherjee et al. [53] found that the diversity of mycobiome in non-smoker HIV-patients was significantly less when compared to the diversity in patients with and without HIV but who were smokers [50]. Regarding genus, Fukui et al. [45] observed that *Candida* and *Malassezia* were the ones that most colonized both groups, with *Penicillium* being the significantly higher gender in the group without HIV [45]. This result contrasts with the result of Mukherjee et al. [44] in which there is a difference in oral mycobiome between HIV-infected and non-infected individuals. In infected individuals, the genus *Candida* was the most prevalent, affecting 92% of this group [44]. Besides that, Mukherjee et al. [44] found that a decrease in *Pichia* is related to an increase in *Candida* [44]. Thus, the results of this study show us that there are changes in oral mycobiome associated with HIV [44]. Mukherjee et al. [53] unlike the other two studies that involved mycobiome sequencing and analysis in patients with and without HIV, compared HIV-infected individuals who were smokers and non-smokers to uninfected smokers. Consequently, they presented different results and verified high levels of *Candida* in individuals with and without HIV who smoked when compared to the non-smoking HIV group [53]. Although the authors of the study did not hypothesize the cause of this reduction, several previous studies have shown that smoking is associated with greater colonization by *Candida* [97–101]. Li et al. [47] and Vijendran et al. [46] did not use next-generation sequencing techniques and identified several fungi in their studies besides *Candida* spp. While Vijendran et al. [46] identified species of *Candida* and filamentous fungi such as *Dermatophyte, Pityrosporum,* and non-dermatophyte filamentous fungi; Li et al. [47] found *C. albicans, C. glabrata, C. parapsilosis, C. krusei, C. tropicalis, C. rugosa, C. norvegensis, C. lusitaniae, C. guilliermondii, Pichia ohmeri,* and *Saccharomyces cerevisiae*. Li et al. [47] performed colorimetric and morphological identification for *Candida* species using CHROMagar® and then used fermentation and assimilation tests through the API 20 C AUX system (bioMérieux, France). In addition, this study also used the chlamydospore production test to differentiate *C. albicans* and *C dubliniensis* that in the selective environment have a similar morphology and coloring [47,92].

Countless techniques are still used as a standard in the identification of fungi among the microscopic aspects. Tests of selective medium, germ tube test, hyphae production and specific medium for the production of chlamydospores are examples of morphological methods. De Mendonça et al. [62], Dos Santos Abrantes et al. [54] and De Mendonça et al. [61] were the three works that only used morphological identification methods. De Mendonça et al. [62] and De Mendonça et al. [61] performed microbiological culture tests to identify *Candida* species present in ALL patients, but did not distinguish between species. The focus of Dos Santos Abrantes et al. [54] was to identify *Candida* species present in HIV-infected patients with oral candidiasis so they used methods for morphological identification through the chromogenic medium followed by gram stain and germ tube test in order to distinguish *C. albicans* from *C. dubliniensis*.

The study by Mushi et al. [73] was the only one that used MS with matrix-assisted ionization and a laser desorption source and time-of-flight analyzer (MALDI-TOF) in the identification and confirmation of non-albicans *Candida* species in HIV patients. This technique facilitates identification when compared to conventional methods because it is faster and has high accuracy for a great variability of microorganisms [102,103]. In addition to microscopic morphological and spectrometric techniques, we can also see that biochemical tests, more precisely the fermentation and assimilation tests, were also widely used in the HIV-infected articles selected in this systematic review (11/21). In 7 articles that studied HIV-infected patients, this test was performed after microbiological culture and morphological identification in order to confirm with greater accuracy the species found [46,47,50,51,55,57,60].

Menezes et al. [59], in addition to using morphological analyses, subsequently performed PCR to distinguish *C. albicans* from *C. dubliniensis* [59], while Thanyasrisung et al. [58] only used PCR for ambiguous results from their biochemical tests [58]. Menezes et al. [59] found 11 different species of *Candida* and nine associations between species of *Candida*, which made it stand out among the others due to the combination of three different identification techniques [59].

Only Mane et al. [70] that studied HIV-infected patients made use of fermentation and assimilation tests using API 20 C AUX (BioMerieux) without any other method of complementary identification [70]. These tests have an accuracy of 92% on average, greater reliability in genus identification and greater discriminatory power when associated with a zymogram [92]. Fermentation and assimilation tests are effective to identify yeasts since they evaluate the ability of a yeast to ferment a certain carbohydrate through the production of carbon dioxide [92].

Among the other molecular techniques, PCR was very present in the identification of species in the selected articles, because of its high degree of sensitivity and selectivity [98, 104]. This technique was used in eight articles, where seven of them analyzed HIV-infected patients [36,44,45,53,56,58,59]. Sharifzadeh et al. [56] and Menezes et al. [59] used specific primers for *C. albicans* and *C. dubliniensis* species, while Moris et al. [52] used other primers for *C. parapsilosis.*

In general, universal primers and amplicon sequencing produce a wide spectra of microbiota. To investigate mycobiota the internal transcribed spacer (ITS) between 18S and 28 S rRna genes is the most used locus [83]. Mukherjee et al. [44], Fukui et al. [45] and Mukherjee et al. [53] amplified the ITS region to detect the presence of several fungi. Javad et al. [36] was the only study that amplified the D1/D2 domain, which identifies yeast species. Thanyasrisung et al. [58] performed PCR using specific primers for five different *Candida* species. This shows that there is a diversity of choice in relation to the region to be amplified by PCR, which makes the study direct its objective, and obtain results faster.

Blignaut and van Heerden [72] was the only work that made use of DGGE which is an excellent tool to assess previously unknown microbial diversity [105]. Using this technique, the authors were able to see that colonization of oral soft tissue and dentin is done by the same genetic subgroup of *C. albicans* [81]. DGGE presents fast and easy analytical methodologies, in addition to separating DNA fragments of the same size, but with different nucleotide sequences [99–101]. The technique has numerous advantages, such as: accessibility in poorly equipped laboratories, screening before sequencing, comparing the efficacy and reproducibility of different DNA extraction protocols and facilitating the interpretation of its results [106–108].

In chronic diseases like AD, *C. albicans* has been shown to be related to the disease, since the production of specific antibodies for *C. albicans*, is associated with the severity of AD, as in the study of Matsumura et al. [105], where IgE antibody levels were significantly higher in patients with AD than in the control group [105]. Javad et al. [36] evaluated antibodies other than IgE such as IgG, IgA and IgM. The authors found statistically significant low levels of IgG for *C. albicans* in AD patients when compared to controls. IgG is an important factor in the humoral immune response and is one of the most important serum immunoglobulins [98]. Thus, we can emphasize that the genus *Candida* is one of the main yeasts that can lead these patients to acquire infections such as oral candidiasis, which may be systematically disseminated by mucositis lesions or when it reaches the oropharynx [19,109].

## Conclusion

Knowledge of the most frequent fungal species in AD, leukemia and HIV is important in order to contribute to the improvement of the clinical condition of the disease, as well as an alert of the patient’s general condition of health. Patients in hospital are at greater risk of acquiring fungal infections, especially those who are immunosuppressed or in critical condition. Therefore, the characterization and monitoring of fungi in hospital environments, of the fungal microbiota on the hands of health professionals and colonized sites of patients are necessary measures to control these microorganisms.

In this systematic review, we found that the genus *Candida* was the most identified among the three diseases studied, with *C. albicans, C. parapsilosis,* and *C. tropicalis* being the species most frequently found. The most frequently identified colonized sites in the selected studies were the oral mucosa and dorsal surface of the tongue. In the field of diagnosis, there is a great diversity of methods used to identify fungi in medical microbiology. However, many of them are still unviable, especially in terms of cost, training of personnel and speed of diagnosis. Despite presenting low precision and only presumptive identification of species, morphological identification by microscopic tests is still widely used in the identification of fungi of patients with leukemia, HIV, and AD. This technique was used before the introduction of biochemical and/or molecular tests that, by providing greater precision, are able to identify species with greater predictability and fewer errors. However, nowadays next-generation sequencing is gradually coming available for these identification tasks and can provide an accurate molecular diagnosis, as well as the quantification of the predominant species in medical microbiology. However, it is not to be seen as a breakthrough for oral fungal identification but rather as a reflection of a shift in the whole field of microbial surveillance.
